# Disparities in Trauma Care for Latino Patients: An Analysis by Trauma Center Verification Level

**DOI:** 10.7759/cureus.76282

**Published:** 2024-12-23

**Authors:** Gabriel Scally, Jesus Orozco, Eva Tovar Hirashima, Nicholas W Sheets

**Affiliations:** 1 General Surgery, HCA Healthcare Riverside, Riverside, USA; 2 School of Medicine, University of California Riverside School of Medicine, Riverside, USA; 3 Emergency Medicine, Riverside Community Hospital, Riverside, USA; 4 Trauma and Acute Care Surgery, Riverside Community Hospital, Riverside, USA

**Keywords:** acute care surgery and trauma, acute trauma care, latino communties, national trauma data bank, racial disparity

## Abstract

Introduction

Trauma is the leading cause of death for individuals under 45 in the United States (US), with significant disparities in outcomes among minority groups. Latinos, the largest ethnic minority in the US, often face barriers to optimal trauma care that may require additional resources. This study aimed to compare trauma outcomes for Latino patients treated at Level I versus Level II/III trauma centers (TCs).

Methods

We conducted a retrospective analysis using the National Trauma Data Bank (NTDB) from 2019 to 2021. Latino patients aged ≥ 18 years treated at Level I and Level II/III TCs were included. The primary outcome was in-hospital mortality, while secondary outcomes included length of stay (LOS), intensive care unit (ICU) LOS, ventilator days, in-hospital complications, and discharge disposition. Propensity score matching was used to control for age, sex, injury severity, and mechanism of injury. Statistical analyses were performed with a significance level of *P* < 0.05.

Results

The unmatched cohort included 221,050 Latino patients, with 139,286 treated at Level I and 81,764 at Level II/III TCs. After matching, 81,764 patients remained in each group. Level I TCs had lower mortality (1.26% vs. 1.48%; *P* < 0.05) and higher discharge-to-home rates (75.22% vs. 73.15%; *P* < 0.05) yet had longer hospital LOS (6.53 ± 0.03 vs. 6.17 ± 0.03; *P* < 0.05), ICU LOS (5.56 ± 0.05 vs. 5.13 ± 0.04; *P* < 0.05), and more in-hospital complications (3.92% vs. 3.67%; *P* < 0.05).

Conclusion

Despite similar baseline characteristics, Latino patients treated at Level I TCs had better survival and disposition outcomes but experienced longer LOS and higher complication rates. While resource availability largely determines trauma verification level and may be responsible for disparities in care, more studies are needed to investigate further how verification level impacts care for Latino patients.

## Introduction

Trauma is the leading cause of death among individuals up to the age of 45 in the United States (US), and disparities in trauma care are well-documented [1]. Studies have shown that minority groups, including the Latino population, face worse outcomes compared to non-Hispanic Whites, with evidence of higher mortality, increased under-triage, and less access to optimal care [2,3]. The Latino population represents the largest ethnic minority group in the US, accounting for more than 19% of the population, and this demographic is projected to continue growing [4]. Therefore, understanding trauma care outcomes in this population is critical for improving health equity.

The American College of Surgeons verification system stratifies trauma centers (TCs) based on their capacity to provide comprehensive care, with Level I TCs being equipped to handle the most severe injuries and Level II and III TCs often handling less critical cases [5]. Prior studies have suggested that patients treated at Level I TCs tend to have better outcomes, although the reasons for these differences remain unclear [5].

Resource availability is largely responsible for TC verification level and may largely affect outcomes in at-risk populations. The Latino population may have unique risk factors necessitating more resource utilization that may be better managed at TCs that are better equipped. Despite being the largest ethnic minority group in the US, research on trauma care outcomes specific to Latino patients remains limited. Prior research has focused predominantly on mortality with less emphasis on other outcomes such as hospital length of stay (LOS), complications, and discharge disposition. Existing studies often aggregate minority groups, failing to analyze outcomes within specific populations which can obscure unique factors and care needs. Our study aims to compare Latino trauma patient outcomes between Level I and Level II/III TCs. We suspect TCs that are known to have more resources may be associated with better outcomes among Latino patients.

## Materials and methods

Study design and data source

This study utilized data from the National Trauma Databank (NTDB), a registry of de-identified trauma demographics, clinical details, and outcomes from TCs across the US. The NTDB is the largest national trauma registry and provides data for examining trauma care outcomes. No institutional review board approval was required due to the de-identified nature of the dataset.

Study population

This study included Latino trauma patients aged ≥ 18 years who presented to TCs across the US from January 1, 2019, to December 31, 2021. Patients were excluded if they lacked TC verification, injury severity score (ISS), age, and sex. TCs were divided into two groups, Level I versus Level II and III, and compared. 

Trauma center classification

TCs were stratified into two groups based on the American College of Surgeons' verification system. Level I TCs are facilities that have the highest level of resources and expertise to manage severe injuries. Level II/III TCs are facilities that handle less critical cases and have fewer resources than level I TCs.

Data collection

Demographics and clinical variables were collected which included demographics (age, sex, and ethnicity), clinical characteristics (mechanism of injury (MOI), vital signs at admission, and ISS. The primary outcome of this study was in-hospital mortality, while secondary outcomes included hospital LOS, intensive care unit (ICU) LOS, ventilator days, in-hospital complications, and discharge disposition.

Statistical analysis

Baseline patient characteristics and outcomes were compared between Level I and Level II/III TCs using Chi-square tests for categorical variables and independent t-tests for continuous variables. To minimize confounding factors, propensity score matching was performed using a 1:1 matching algorithm. Matching variables included age, sex, ISS, and MOI, selected for their known influence on trauma outcomes. After matching, comparisons between groups were conducted using paired t-tests for continuous variables and McNemar's test for categorical variables. A p-value < 0.05 was considered statistically significant. All statistical analyses were performed using RStudio version 2024.04.2 + 764.

## Results

Baseline characteristics of Latino trauma patients

A total of 221,050 Latino trauma patients were included in this analysis, with 139,286 treated at level I TCs and 81,764 at level II/III TCs. The mean age was significantly younger at level I TCs compared to level II/III TCs (44.61 + 0.05 vs. 46.04 + 0.07 years; *P* < 0.05) (Table 1). The majority of patients were male at all TCs. The proportion of blunt trauma was significantly higher at level II/III TCs (70,309 (85.99%) vs. 115,593 (82.99%); *P* < 0.05), while penetrating trauma was more frequent at level I TCs (19,639 (14.10%) vs. 10,081 (12.33%); *P* < 0.05) (Table 1). The ISS was significantly higher at level I TCs than at level II/III TCs (10.53 + 0.03 vs. 9.98 + 0.03; *P* < 0.05) (Table 1).

Comparison of outcomes between level I and level II/III TCs (unmatched)

Mortality rates were comparable between the groups, with 2,103 (1.51%) at level I TCs and 1,210 (1.48%) at level II/III TCs (Table 1). However, the rate of discharge to home was higher for patients treated at level I TCs (104,868 (75.29%) vs. 59,810 (73.15%); *P* < 0.05) (Table 1). Hospital LOS (6.84 + 0.03 vs. 6.17 + 0.03; *P* < 0.05), ICU LOS (5.91 + 0.04 vs. 5.13 + 0.04; *P* < 0.05), and ventilator days (6.54 + 0.07 vs. 6.03 + 0.09; *P* < 0.05) were all significantly longer at level I TCs (Table 1). In-hospital complications were also more frequent at level I TCs (5,794 (4.16%) vs. 3,001 (3.67%); *P* < 0.05) (Table 1).

Propensity score matching analysis

After propensity score matching, 81,764 patients remained in each group, with no significant differences in mean age or ISS. However, some disparities persisted: blunt trauma remained higher at level II/III TCs (70,309 (85.99%) vs. 68,257 (83.48%); *P* < 0.05), while penetrating trauma was more common at level I TCs (10,809 (13.22%) vs. 10,081 (12.33%); *P* < 0.05) (Table 2).

Comparison of outcomes between level I and level II/III TCs (matched)

Level I TCs showed higher rates of in-hospital complications (3,205 (3.92%) vs. 3,001 (3.67%); *P* < 0.05), hospital LOS (6.53 + 0.03 vs. 6.17 + 0.03; *P* < 0.05), and ICU LOS (5.56 + 0.05 vs. 5.13 + 0.04; *P* < 0.05) (Table 2). Mortality was lower at level I TCs post-matching 1,030 (1.26%) vs. 1,210 (1.48%); *P* < 0.05) (Table 2), and patients treated at level I TCs were more frequently discharged to home (61,503 (75.22%) vs. 59,810 (73.15%); *P* < 0.05) (Table 2).

**Table 1:** Unmatched demographics and outcomes of Latino trauma patients between level I and level II/III trauma centers LOS: length of stay The data is presented as N (%) or mean ± standard error (SE), as appropriate. A *P*-value of <0.05 was considered statistically significant. Continuous variables were analyzed using a t-test^a^, while categorical variables were analyzed using a chi-square test^b^

	Total patients	Level I	Level II & III	*P-*value
N	221050	139286 (63.01%)	81764 (36.99%)	
Age	45.12 (±0.04)	44.61 (±0.05)	46.04 (±0.07)	<0.001^a^
Male	156393 (70.75%)	99631 (71.53%)	56769 (69.43%)	<0.001^b^
Mechanism				
Blunt	185903 (84.10%)	115593 (82.99%)	70309 (85.99%)	<0.001^b^
Penetrating	29731 (13.45%)	19639 (14.10%)	10081 (12.33%)	<0.001^b^
ISS	10.33 (±0.02)	10.53 (±0.03)	9.98 (±0.03)	<0.001^a^
Pulse	89.30 (±0.05)	89.38 (±0.06)	89.18 (±0.08)	0.04^a^
Respiratory rate	18.76 (± 0.01)	18.69 (±0.01)	18.89 (±0.02)	<0.001^a^
Systolic blood pressure	135.95 (± 0.09)	135.14 (±0.08)	137.35 (±0.10)	<0.001^a^
Outcomes				
In-hospital complication	8798 (3.98%)	5794 (4.16%)	3001 (3.67%)	<0.001^b^
Hospital LOS	6.59 (±0.02)	6.84 (±0.03)	6.17 (±0.03)	<0.001^a^
ICU LOS	5.62 (±0.03)	5.91 (±0.04)	5.13 (±0.04)	<0.001^a^
Ventilator days	6.36 (±0.05)	6.54 (±0.07)	6.03 (±0.09)	<0.001^a^
Mortality	3316 (1.50%)	2103 (1.51%)	1210 (1.48%)	0.65^b^
Disposition to home	164682 (74.50%)	104868 (75.29%)	59810 (73.15%)	<0.001^b^

**Table 2:** Matched demographics and outcomes of Latino trauma patients between level I and level II/III trauma centers LOS: length of stay The data is presented as N (%) or mean ± standard error (SE), as appropriate. A *P*-value of <0.05 was considered statistically significant. Continuous variables were analyzed using a paired t-test^a^, while categorical variables were analyzed using McNemar’s test^b^

	Total patients	Level I	Level II & III	*P-*value
N	163528	81764 (50.00%)	81764 (50.00%)	
Age	46.0 (±0.05)	45.97 (±0.07)	46.04 (±0.07)	0.53^a^
Male	113799 (69.59%)	57039 (69.76%)	56769 (69.43%)	0.01^b^
Mechanism				
Blunt	138574 (84.74%)	68257 (83.48%)	70309 (85.99%)	<0.001^b^
Penetrating	20882 (12.77%)	10809 (13.22%)	10081 (12.33%)	<0.001^b^
ISS	9.95 (±0.02)	9.92 (±0.03)	9.98 (±0.03)	0.14^a^
Pulse	89.07 (±0.05)	88.97 (±0.08)	89.18 (±0.08)	0.06^a^
Respiratory rate	18.74 (±0.01)	18.60 (±0.02)	18.89 (±0.02)	<0.001^a^
Systolic blood pressure	136.71 (±0.04)	136.07 (±0.10)	137.35 (±0.10)	<0.001^a^
Outcomes				
In-hospital complication	6198 (3.79%)	3205 (3.92%)	3001 (3.67%)	0.01^b^
Hospital LOS	6.35 (±0.02)	6.53 (±0.03)	6.17 (±0.03)	<0.001^a^
ICU LOS	5.34 (±0.03)	5.56 (±0.05)	5.13 (±0.04)	<0.001^a^
Ventilator days	6.09 (±0.06)	6.16 (±0.09)	6.03 (±0.09)	0.32^a^
Mortality	2240 (1.37%)	1030 (1.26%)	1210 (1.48%)	<0.001^b^
Disposition to home	121321 (74.19%)	61503 (75.22%)	59810 (73.15%)	<0.001^b^

## Discussion

This study reveals significant disparities in trauma care outcomes for Latino patients treated at level I versus level II/III TCs in the US. Even after controlling for key demographics and injury severity, we observed differences in MOI, in-hospital complications, hospital LOS, ICU LOS, mortality, and disposition between TC levels. These findings have implications for trauma system design and care delivery as they relate to the outcomes of Latino patients.

One finding from this study is the lower mortality rate observed at level I TCs. While overall mortality was low in both groups, the relative reduction at level I TCs, compared to level II/III TCs, was significant. This finding aligns with previous research demonstrating that patients treated at higher-level TCs have better survival rates [6]. Level I TCs are typically equipped with more comprehensive resources, including specialized surgical teams, advanced diagnostic tools, and the ability to provide 24-hour critical care, ultimately leading to improved survival. While level II/III TCs are designed to handle trauma cases, they lack the full spectrum of resources available at level I TCs. For Latino patients, who often face barriers such as limited access to healthcare, socioeconomic challenges, and potential language barriers, the limitations of level II/III TCs may exacerbate existing disparities [7,8]. Castro et al. found that trauma patients with limited English proficiency were associated with increased mortality [9]. These findings suggest a need for targeted improvements in the capabilities of level II/III TCs, particularly in regions with a high density of Latino populations.

Although level I TCs were associated with lower mortality, they exhibited longer hospital and ICU LOS. This finding likely reflects the greater complexity and severity of cases managed at these facilities due to multisystem injuries requiring prolonged care and advanced surgical intervention with extended postoperative monitoring. While prolonged stays may increase resource utilization and healthcare costs, they may also contribute to better long-term outcomes. In contrast, patients treated at level II/III TCs may be discharged earlier, potentially without receiving the same level of follow-up care, which could negatively affect recovery and long-term health outcomes. It is also worth considering that the longer LOS at the level I TCs may be influenced by the availability of specialized rehabilitation services, which is crucial for improving functional outcomes in trauma patients [10]. Level I TCs, which often have established relationships with rehabilitation facilities, may be better equipped to coordinate these services, whereas level II/III TCs may discharge patients earlier due to resource limitations. 

Our study also found significant differences in disposition, with a higher proportion of patients treated at level I TCs being discharged to home. Discharge to home is an important outcome metric, as it indicates that patients have recovered sufficiently to return to their pre-injury environment without the need for long-term rehabilitation. In contrast, the lower rate of discharge to home at level II/III TCs may reflect gaps in post-acute care planning. Moreover, discharge to a facility other than the home can increase the risk of readmission and can lead to worse long-term outcomes [11]. For Latino patients, who may already face barriers to accessing follow-up care due to socioeconomic or geographic factors, these disparities in disposition are particularly concerning. 

The observed disparities in outcomes between level I and level II/III TCs have important implications for health equity. Latino populations often experience barriers to healthcare access, including insurance coverage issues, language barriers, and geographic disparities [7,8]. These barriers are particularly pronounced in trauma care, where timely access to a well-equipped facility can mean the difference between life and death. Strengthening the capabilities of level II/III TCs, particularly in areas with large Latino populations, should be a priority for healthcare policymakers. This may include expanding resources and enhancing coordination between TCs to facilitate timely transfers when necessary. In addition to improving the capacity of TCs, culturally competent care that addresses the needs of Latino patients is needed. This includes ensuring language services are available, providing education on cultural differences, and recognizing social determinants of health that may affect outcomes. Addressing these factors is essential to closing the gap in trauma care outcomes.

Despite the large sample size and the use of propensity score matching, this study has several limitations. First, the retrospective nature of the data limits our ability to draw causal inferences about the relationship between TC level and outcomes. While we controlled for key variables, other important factors such as comorbidities, pre-existing social conditions, and the availability of rehabilitation services were not included. Additionally, the NTDB relies on reporting by TCs, where coding errors or variability in data collection could introduce bias. While we attempted to match patients based on important covariates, there may still be unmeasured differences between level I and level II/III TCs that could affect outcomes.

While this study focused on Latino patients, the NTDB does not capture information on subgroups within this population. The Latino population represents diverse backgrounds from different countries, cultures, and regions. Future research should consider examining factors such as immigration status, language proficiency, cultural background, and other social determinants of health that may influence outcomes. Understanding the role of prehospital care, transfer protocols, and post-discharge care is essential. Intervention-based studies targeting improvements in care coordination between level I and II/III TCs could also provide valuable insights. Lastly, efforts to improve the cultural competency of trauma care providers should be prioritized. Ensuring that TCs have the tools and training necessary to provide equitable, patient-centered care for Latino populations is crucial in improving health outcomes for this growing demographic. 

## Conclusions

This study demonstrates that while level I TCs provide superior outcomes for Latino trauma patients, disparities persist between TC levels, particularly in terms of mortality, LOS, and discharge disposition. Addressing these gaps requires targeted investments to enhance resources and staffing at lower-level TCs, standardized care pathways for managing severe injuries, and training in culturally competent care. Strengthening regional trauma networks and improving interhospital transfers can also ensure timely escalation of care. Additionally, trauma systems should monitor disparities through stratified reporting of outcomes to drive quality improvement initiatives. These measures can help reduce disparities and improve outcomes for minority trauma patients, including the Latino population, advancing equity in trauma care.
